# Case Report: Extracorporeal photopheresis for cutaneous lupus erythematosus induces putatively atheroprotective B and T cell responses

**DOI:** 10.3389/fimmu.2026.1741656

**Published:** 2026-01-30

**Authors:** Marcella Visentini, Maria Ozsvar-Kozma, Ilenia Pacella, Alessandra Pinzon Grimaldos, Federica Falco, Mahnaz Shafii-Bafti, Ilenia Minicocci, Francesca La Gualana, Maurizio Carlesimo, Milvia Casato, Stefania Basili, Marcello Arca, Silvia Piconese, Massimo Fiorilli, Christoph J. Binder

**Affiliations:** 1 Division of Clinical Immunology, Department of Translational and Precision Medicine, Sapienza University of Rome, Rome, Italy; 2 Laboratory affiliated to Istituto Pasteur Italia-Fondazione Cenci Bolognetti, Department of Translational and Precision Medicine, Sapienza University of Rome, Rome, Italy; 3 Department of Laboratory Medicine, Medical University of Vienna, Vienna, Austria; 4 Department of Surgery, Sapienza University of Rome, Rome, Italy; 5 Laboratory of Immune Regulation, Department of Translational and Precision Medicine, Sapienza University of Rome, Rome, Italy; 6 Immunohematology and Transfusion Medicine, Department of Translational and Precision Medicine, Sapienza University of Rome, Rome, Italy; 7 Division of Metabolic Diseases, Department of Translational and Precision Medicine, Sapienza University of Rome, Rome, Italy

**Keywords:** anti-OxLDL antibodies, atheroprotection, atherosclerosis, cutaneous lupus erythematosus, extracorporeal photopheresis, regulatory T cells

## Abstract

Extracorporeal photopheresis (ECP) involves the reinfusion of autologous peripheral blood lymphocytes rendered apoptotic by *in vitro* exposure to psoralen and ultraviolet A light. Antigenic determinants presented by apoptotic lymphocytes, primarily T cells, elicit immunomodulatory responses that have shown therapeutic benefit in several conditions, including cutaneous T-cell lymphoma, graft-versus-host disease, and various inflammatory/autoimmune disorders. We treated with ECP a 41-year-old woman diagnosed with cutaneous lupus erythematosus and concomitant hypercholesterolemia, achieving a marked improvement of skin lesions. A study in hypercholesterolemic apolipoprotein E-deficient mice demonstrated that immunization with syngeneic apoptotic thymocytes, a process mimicking ECP, induced the production of IgM antibodies against oxidized low-density lipoproteins (OxLDL) that attenuated atherosclerosis. Thus, we explored whether ECP could similarly induce anti-OxLDL antibodies in our patient. Indeed, over the course of a 14-week ECP treatment we observed a steady increase in circulating IgM antibodies against malondialdehyde-modified LDL, a class of antibodies known to confer atheroprotection in preclinical models. Additionally, we documented an increase in circulating regulatory T cells, which are recognized as suppressing pro-atherogenic immune responses. These findings support the translational potential of a preclinical atheroprotection model and provide a proof of concept for clinical trials evaluating ECP in autoimmune diseases associated with accelerated atherosclerosis, where achieving dual benefits, clinical improvement and reduced cardiovascular risk, may be feasible.

## 1 Introduction

Extracorporeal photopheresis (ECP) is an immunomodulatory therapy consisting of repeated injection of autologous peripheral blood leukocytes treated *ex vivo* with psoralen, a photosensitizer agent, and then exposed to ultraviolet A light. This treatment induces lymphocyte apoptosis, substantially limited to T cells, as well as the generation of monocyte-derived dendritic cells able to present immunogenic neoepitopes of apoptotic cells (1, 2). The immunomodulatory mechanisms of action of ECP are incompletely understood (3, 4); they involve an increase in anti-inflammatory cytokines such as IL-10 and TGF-β (5) and an increase of CD4^+^FoxP3^+^ regulatory T cells (Tregs) that can suppress effector T cells and reverse experimental graft-versus-host disease (6) or contact hypersensitivity (7).

ECP, which has demonstrated an excellent safety profile over several decades, it is approved by the U.S. Food and Drug Administration as first line therapy for erythrodermic cutaneous T-cell lymphoma, it is highly effective in managing graft-versus-host disease and has shown significant clinical benefits in allograft rejection and in several autoimmune disorders (8–10).

Based on previous favorable reports (10), we treated with ECP a patient with steroid-dependent cutaneous lupus erythematosus and obtained a rather good clinical outcome. A preclinical study (11) had shown that immunization with syngeneic apoptotic thymocytes, a procedure reminiscent of ECP, induced in apolipoprotein E-deficient (*Apoe^−/−^*) mice the production of IgM antibodies against oxidated low-density lipoproteins (OxLDL) and protection from atherosclerosis. Apoptotic cells expose the same oxidation-specific epitopes that are also present on OxLDL, providing an explanation for the expansion of these antibodies in this setting (12). This study prompted us to investigate in this patient, who concomitantly had hypercholesterolemia, whether ECP could similarly elicit potentially atheroprotective immune responses, namely IgM antibodies against OxLDL and regulatory T cells (Tregs) (13).

## 2 Methods

The ECP treatment protocol consisted of 4 weekly sessions, followed by 3 sessions every two weeks and then by monthly sessions as maintenance. Further details are provided in **Supplementary Information**.

We used in-house chemiluminescent ELISAs to measure IgM and IgG antibodies to human OxLDL, namely malondialdehyde-modified low-density lipoprotein (MDA-LDL), copper–oxidized LDL (Cu-OxLDL), and phosphorylcholine-modified bovine serum albumin (PC-BSA) in serum as previously described (14). Measurements are expressed as Relative Light Units per 100 milliseconds (RLU/100ms). Tregs were identified by flow cytometry as CD3^+^CD4^+^CD127^low^FOXP3^+^ cells, as previously described (15). Tregs were further subdivided into three distinct subsets: FOXP3^low^CD45RA^+^ resting Tregs, FOXP3^high^CD45RA^-^ activated Tregs, and FOXP3^low^CD45RA^-^ non-Tregs; the latter cells constitute a non-suppressive subset of Tregs that is expanded in systemic lupus erythematosus (16, 17). Further details are provided in **Supplementary Information**.

## 3 Case report

This 41-year-old woman was diagnosed with discoid lupus erythematosus at the age of 24; she also had hypercholesterolemia. Over years, skin lesions worsened and new inflamed and painful lesions appeared on the trunk despite treatment. Hydroxychloroquine and cyclosporine were poorly effective and frequent courses of systemic corticosteroids were needed for controlling disease activity; for these reasons ECP therapy was proposed. At the start of ECP treatment, she had been on daily prednisone (0.5 mg/kg) for three weeks and presented with inflamed lesions on the face and trunk. During the subsequent clinical course, a marked improvement in cutaneous lesions was observed (**Supplementary Figure S1**), permitting the discontinuation of systemic corticosteroid therapy after four months, while ECP was continued as maintenance therapy. At 11 months, the Cutaneous Lupus Erythematosus Disease Area and Severity Index (CLASI) (18) score had decreased from 41 (activity) and 19 (damage) to 26 and 13, respectively, a change indicative of a clinically meaningful reduction in disease activity (19). She continued monthly ECP sessions without the need of significant systemic corticosteroid therapy for over two years, with stable cutaneous lupus activity ad damage; no treatment-related adverse events were observed. Further information on the patient’s clinical course is provided in **Supplementary Material**.

## 4 Atheroprotective immune responses elicited by ECP

**Figure 1:**
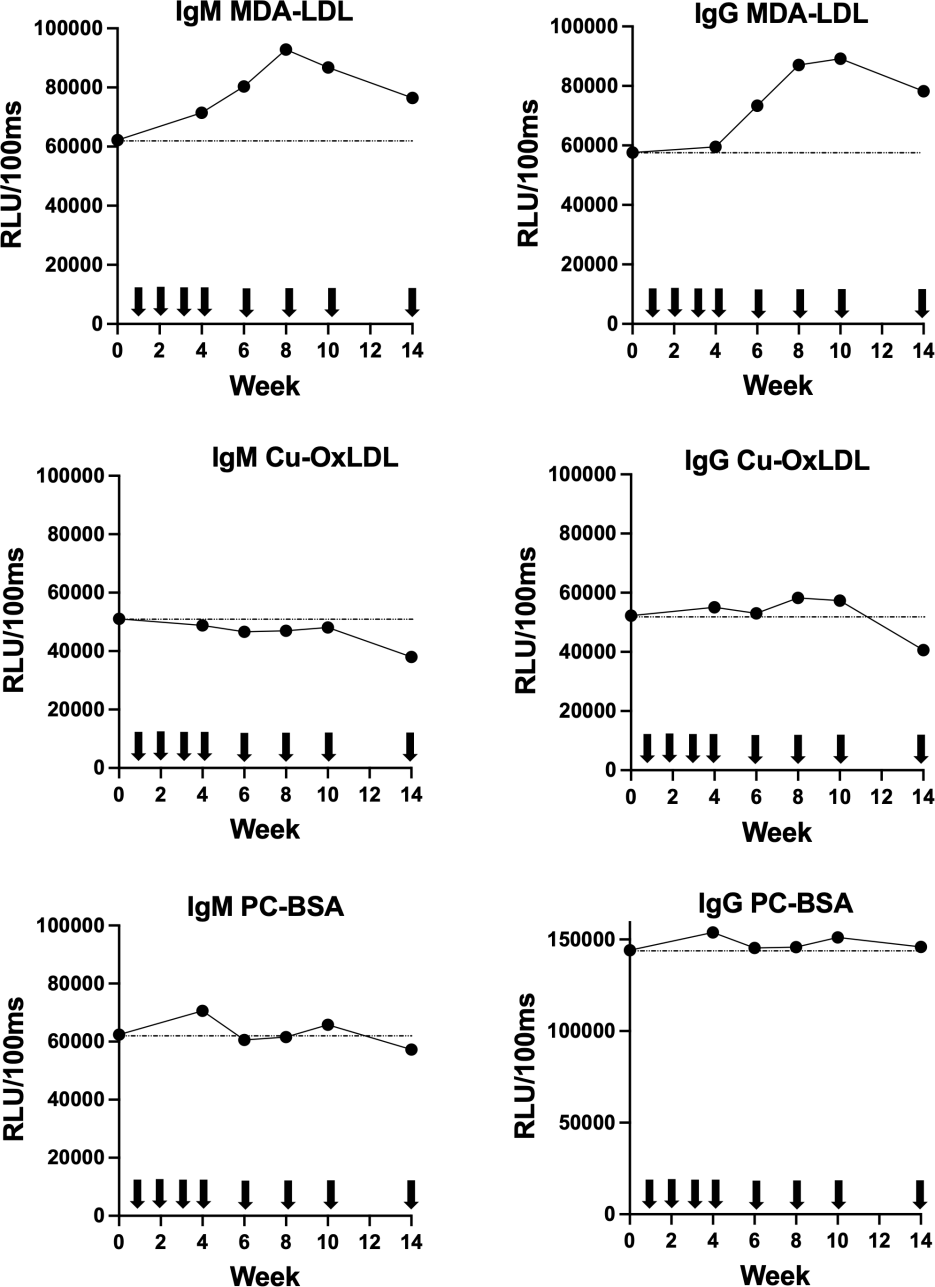
Longitudinal changes during 14 weeks of ECP therapy in chemiluminescent ELISA titers of serum IgM and IgG antibodies against distinct human LDL (MDA-LDL, Cu-OxLDL, and PC-BSA). RLU, Relative Light Units per 100 milliseconds. Arrows denote ECP sessions.

IgM and IgG antibody levels against MDA-LDL, CuOx-LDL, and PC-BSA were repeatedly measured from baseline through week 14 of ECP therapy in the patient with cutaneous lupus erythematosus and hypercholesterolemia (**Figure 1**). Antibody titers to CuOx-LDL and PC-BSA remained largely unchanged over the course of treatment. In contrast, a progressive increase in both IgM and IgG antibodies targeting MDA-LDL was observed in response to ECP.

**Figure 2:**
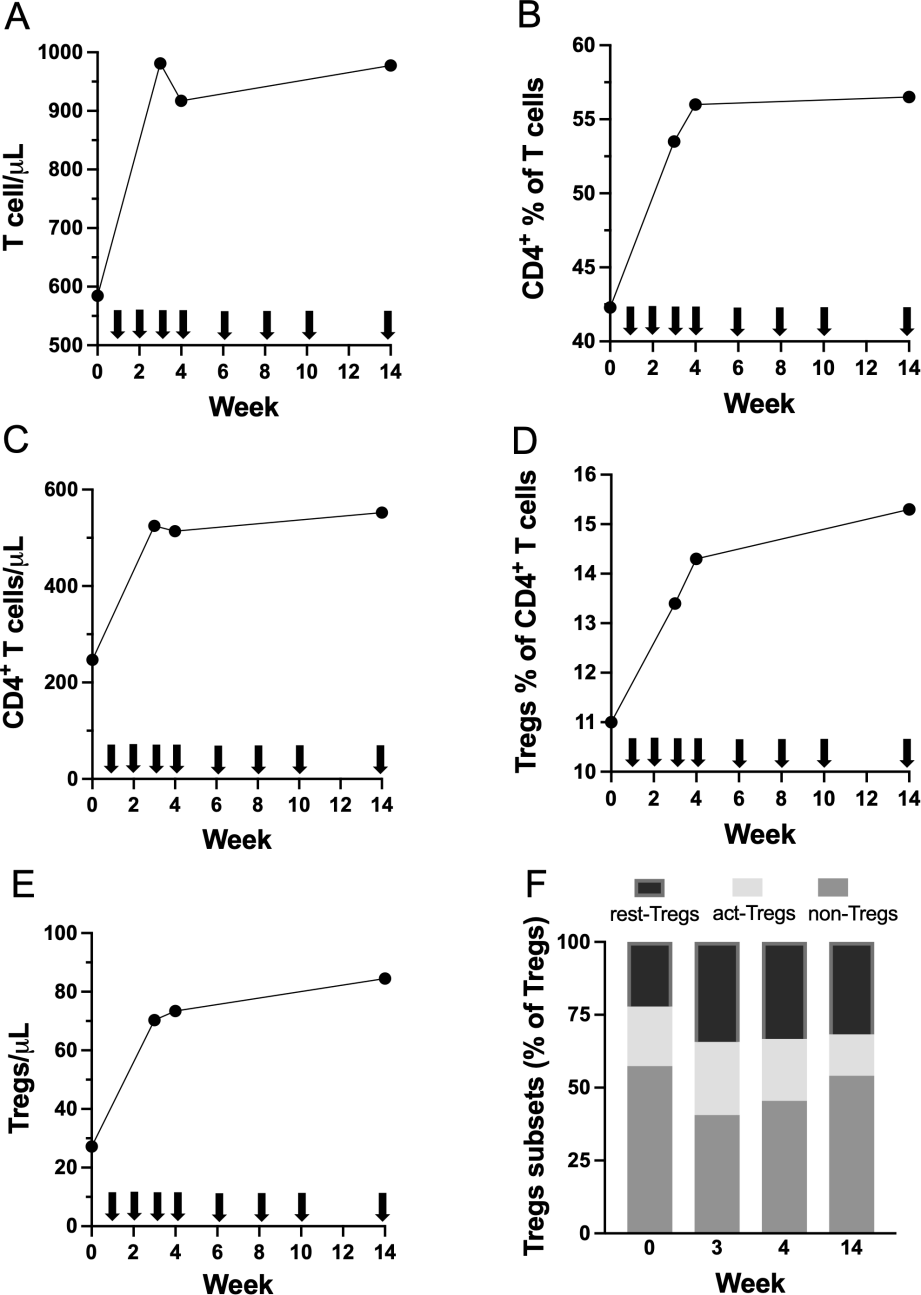
Longitudinal changes during 14 weeks of ECP therapy in **(A)** absolute count of total T cells; **(B)** percentage among T cells and **(C)** absolute count of CD4^+^ T cells; **(C)** percentage among T cells and **(D)** absolute count of total Tregs; **(F)** relative proportions of Tregs subsets (resting-Tregs, activated-Tregs, and non-Tregs). Arrows denote ECP sessions.

ECP treatment was associated with an increase in the absolute T cell count (**Figure 2A**), along with an increase in the percentage among T cells (**Figure 2B**) and in the absolute count (**Figure 2C**) of CD4^+^ T cells. Importantly, a progressive rise in both the proportion among CD4^+^ T cells (**Figure 2D**) and in the absolute count (**Figure 2E**) of circulating Tregs were observed. Moderate changes were observed in the relative proportions of Treg subsets, with a trend toward an increase of resting Tregs and a decrease of non-Tregs (**Figure 2F**). The study in *Apoe*^-/-^ mice reported a significant reduction in total serum cholesterol after four weekly injections of apoptotic thymocytes (11); in our patient, we observed >10% reduction in total and LDL cholesterol and in other proatherogenic lipids and lipoproteins after four weekly ECP sessions (**Supplementary Table S1**).

## 5 Discussion

Our cases strengthen the currently limited body of evidence (10) supporting the efficacy and safety of ECP in cutaneous lupus erythematosus. More interestingly, we show in a patient with hypercholesterolemia that ECP can induce the generation of anti-OxLDL antibodies and of Tregs, both acknowledged as having antiatherogenic effects.

Oxidative stress, arising from various sources, induces lipid peroxidation and leads to the formation of oxidation-specific epitopes (OSEs), as neo-self epitopes on the membranes of apoptotic cells and on lipoproteins; this process serves a physiological role in the clearance of dying cells but also contributes to chronic inflammation and atherosclerosis (20). In fact, OxLDL accumulates within the vessel wall, promoting inflammation, foam cell formation, and other pathological processes that drive plaque development and the progression of atherosclerosis (21). The pathogenic effects of OxLDL can be mitigated by natural IgM antibodies that are produced by innate-like B cells in the spleen. Notably, approximately 30% of natural IgM antibodies target OSEs (14), and high levels of OSEs-specific IgM antibodies are associated with lower risk of cardiovascular disease (22).

Several preclinical mouse models have demonstrated that immunization with OSEs can enhance the production of natural IgM antibodies against OxLDL to levels sufficient to confer protection against the progression of atherosclerosis. The first evidence of this was provided by studies showing that immunization with *Streptococcus pneumoniae* induced natural IgM antibodies targeting phosphorylcholine moieties present on the bacterial capsular polysaccharide. These antibodies cross-reacted with OxLDL and attenuated atherosclerosis development in LDL receptor-deficient (*LDLR^–/-^*) mice (23). Similarly, immunization with cell-free phosphorylcholine elicited atheroprotective anti-OxLDL IgM antibodies in *Apoe^–/–^* mice (24).

Murine apoptotic thymocytes contain immunodominant OSEs that elicit robust IgM responses targeting OxLDL (Cu-OxLDL), phosphorylcholine, and MDA-LDL (25). In contrast, injection of apoptotic autologous lymphocytes through ECP triggered in our patient IgM and IgG responses against MDA-LDL without inducing reactivity to Cu-OxLDL or phosphorylcholine. This discrepancy might be due to differences in the mechanisms of action of dexamethasone, used to induce apoptosis in mouse models (11, 25), and of ECP; for example, psoralen directly induces photooxidation of proteins and lipids (26, 27). Untangling this hypothesis requires preclinical studies comparing the immunological effects of apoptotic thymocytes generated either by dexamethasone or by a procedure exactly mimicking human ECP.

Here, we demonstrate that ECP induces a robust IgM response against MDA-LDL, a class of antibodies previously shown to exert atheroprotective effects in murine models. A limitation of this exploratory study is that immunological responses were assessed only during the induction phase of ECP therapy (14 weeks), which roughly mirrors the duration of some preclinical anti-atherogenic immunization protocols. Consequently, we were unable to determine whether the observed increase in anti–MDA-LDL antibody levels was sustained during the subsequent maintenance phase of monthly ECP treatments.

In addition, we observed an expansion of regulatory T cells (Tregs), which have also been implicated in protection against atherosclerosis in preclinical studies. However, newly generated Tregs appear to migrate inefficiently from the periphery to atherosclerotic plaques and therefore may not effectively attenuate disease progression unless specifically engineered to express appropriate chemokine receptors (28). Nevertheless, both intraperitoneal administration of ex vivo–expanded, ovalbumin-specific Tregs (29) and systemic Treg expansion achieved through treatment with an interleukin (IL)-2/anti–IL-2 antibody complex (30) have been shown to significantly reduce atherosclerotic lesion size in *Apoe^−/−^* mice.

MDA is a reactive aldehyde generated during *in vivo* lipid peroxidation, capable of modifying LDL to form MDA-LDL adducts. Immunization with MDA-modified LDL has been shown to confer protection against atherosclerosis in *LDLR*^⁻/⁻^ rabbits and mice (31, 32), and ongoing efforts aim to identify immunodominant MDA-derived epitopes suitable for the development of vaccines designed to mitigate atherosclerosis (33). The interaction between MDA and lysine residues results in the formation of malondialdehyde-acetaldehyde (MAA) adducts, which are fluorescent and contain immunogenic epitopes that elicit atheroprotective antibody responses against both MDA and MAA haptens (33). Importantly, MAA epitopes are abundantly expressed on the surface of apoptotic cells (34), and approximately 25% of natural IgM antibodies present in murine plasma and human cord blood specifically recognize MAA epitopes (14, 33). These observations support the hypothesis that IgM antibodies targeting MDA/MAA haptens induced by the injection of apoptotic lymphoid cells, generated through ECP, may represent an effective anti-atherogenic immune response in humans.

## 6 Conclusion

Our findings provide proof of concept for the translational relevance of a preclinical mouse model (11) of anti-atherogenic immunization using syngeneic apoptotic lymphoid cells. ECP has yielded promising results in the treatment of chronic autoimmune and inflammatory diseases associated with accelerated atherosclerosis and increased cardiovascular risk (35–39). Upcoming confirmatory clinical trials in these settings are expected, offering an opportunity to further explore and conclusively establish the potential anti-atherogenic effects of ECP.

## Data Availability

The original contributions presented in the study are included in the article/**Supplementary Material**. Further inquiries can be directed to the corresponding author.
