# Supplementary material for: Case Report: Extracorporeal photopheresis for cutaneous lupus erythematosus induces putatively atheroprotective B and T cell responses
Source: Front Immunol. 2026 Jan 30;17:1741656. doi: 10.3389/fimmu.2026.1741656 (PMC12901466; doi:10.3389/fimmu.2026.1741656)
Supplement: Supplementary file 1 [file DataSheet1.pdf]

Supplementary Material

1 Supplementary Methods

1.1 ECP protocol

An ECP session involved the apheresis-based collection of patient's peripheral blood mononuclear cells (MNCs) using a Haemonetics MCS+ cell separator. Red blood cells and plasma were returned to the patient, while the collected MNC fraction was incubated with 8-methoxypsoralen, a photoactivatable DNA-intercalating agent. The cells were subsequently exposed to ultraviolet A (UVA) irradiation to induce DNA damage and apoptosis, after which the treated MNCs were reinfused into the patient. One total blood volume was processed in each session. Published ECP schedules are highly variable depending on centers and on the diseases treated (1,2); the ECP schedule used in this patient was based on our center's experience and consisted of 4 weekly sessions, followed by 3 sessions every two weeks and then by monthly sessions as maintenance.

1.2 Measurement of anti-OxLDL antibodies

Antibodies to MDA-LDL, CuOx-LDL, and PC-BSA were measured by chemiluminescent ELISA as describe before (3,4). In brief, antigens were coated on ELISA plates at 5µg/ml in PBS, washed and blocked, and sera diluted between 1:200 and 1:800 in TBS/EDTA containing BSA were added. Bound antibodies were detected using alkaline phosphatase-labeled goat-anti-human IgG or IgM followed by chemiluminescent detection with Lumiphos plus using a chemiluminescent reader. Data are presented as relative light units (RLU) per 100 ms and represent triplicate determinations.

1.3 Enumeration of Treg cells through flow cytometry

Whole blood was collected and analyzed before treatment and after 3, 4 and 14 weeks. At each time point, 100 µl of whole blood were stained in a 12 x 75 mm round-bottom tube as described below. First, 1 µl of undiluted Fixable Viability Dye eFluor 780 (eBioscience Thermo Fisher Scientific) was added to whole blood. Then, a cocktail with the following antibodies was added and incubated 30 minutes at room temperature in the dark: CD3 Alexa Fluor 488 (BioLegend), CD45RA PE (BioLegend), CD127 Pe-Cy7 (BioLegend), CD8 APC-Cy7 (BioLegend), CD14, CD16, CD19 and CD56 APC-eFluor 780 (all from eBioscience Thermo Fisher Scientific), CD25 Brilliant Violet 421 (BioLegend), and CD4 Brilliant Violet 510 (BioLegend). Erythrocytes were lysed by adding 2 ml of BD FACS Lysing Solution (BD Biosciences). After washing, cells were fixed and permeabilized and intracellular staining with FOXP3 PerCP-Cy5.5 (eBioscience Thermo Fisher Scientific) was performed, using the FOXP3/Transcription Factor Staining Buffer Set according to the manufacturer's instructions (eBioscience Thermo Fisher Scientific). All stains were done in duplicate.

Data were acquired on an LSR Fortessa cell analyzer (Becton Dickinson) and analyzed with FlowJo software (version 10.7.1; BD Biosciences). For the gating strategy, lymphocytes were selected based on SSC-A and FSC-A, doublets excluded using FSC-A versus FSC-W and SSC-A versus SSC-W gates. CD4 T cells were gated as live cells staining negative for dump gate markers identifying non-T-cell lineages (CD14, CD16, CD19 and CD56) and negative for CD8.

References

1) Knobler R, Arenberger P, Arun A, Assaf C, Bagot M, Berlin G, et al. European dermatology forum: Updated guidelines on the use of extracorporeal photopheresis 2020 - Part 1. J Eur Acad Dermatol Venereol (2020) 34(12):2693-716. doi: 10.1111/jdv.16890
2) Asensi Cantó P, Sanz Caballer J, Solves Alcaína P, de la Rubia Comos J, Gómez Seguí I. Extracorporeal Photopheresis in Graft-versus-Host Disease. Transplant Cell Ther (2023) 29(9):556-66. doi: 10.1016/j.jtct.2023.07.001.

3) Chou MY, Fogelstrand L, Hartvigsen K, Hansen LF, Woelkers D, Shaw PX, et al. Oxidation-specific epitopes are dominant targets of innate natural antibodies in mice and humans. J Clin Invest (2009) 119(5):1335-49. doi: 10.1172/JCI36800
4) Amir S, Hartvigsen K, Gonen A, Leibundgut G, Que X, Jensen-Jarolim E, et al. Peptide mimotopes of malondialdehyde epitopes for clinical applications in cardiovascular disease. J Lipid Res (2012) 53(7):1316-26. doi: 10.1194/jlr.M025445

2 Case report

This 41-year-old woman had no family history of autoimmune disorders. However, several first- and second-degree relatives had experienced myocardial infarctions around the age of 50 and/or had elevated cholesterol levels. She herself had persistently high cholesterol levels and was not receiving any lipid-lowering therapy at the time of the study. A carotid ultrasound performed at the initiation of ECP therapy showed normal intima-media thickness without other abnormalities.

At the age of 24, she developed erythematous, scaly plaques on her face. A skin biopsy revealed immunoglobulin and complement deposition at the dermal-epidermal junction. Antinuclear antibodies were positive at low titers (1:160), while anti-dsDNA and anti-extractable nuclear antigen antibodies were negative. Based on these findings, a diagnosis of discoid lupus erythematosus was established.

Over the following six years, she was treated with hydroxychloroquine, topical corticosteroids, and occasional short courses of systemic corticosteroids, achieving good control of her skin manifestations. Subsequently, her facial lesions worsened, and new inflamed, painful lesions appeared on her trunk. The cutaneous disease responded only moderately to topical corticosteroids and hydroxychloroquine. Control of disease activity required repeated courses of systemic corticosteroids at low to moderate doses (e.g., prednisone 10–25 mg/day) over the subsequent years. A four-month trial of cyclosporine at 3.5 mg/kg/day was unsuccessful, as flares of inflammation and pain persisted during treatment. Given the limited efficacy of immunosuppressive therapy and hydroxychloroquine, and the need for recurrent systemic corticosteroids to control disease activity, a trial of ECP was proposed. The patient provided informed consent for ECP treatment and participation in additional research studies.

During the first five months of ECP therapy, she experienced a marked improvement in skin lesions, which became less inflamed, non-painful, and non-pruritic (Supplementary Figure 1). However, after missing a scheduled monthly ECP session due to personal constraints, she experienced a disease flare characterized by the appearance of new inflamed and painful lesions. This flare was managed with a short course of corticosteroids, and ECP frequency was increased to a session every 2 weeks for three months followed by monthly sessions. She continued ECP monthly sessions for over 24 months, without treatment-related adverse events and with stable cutaneous lupus activity and damage.

3 Supplementary Figures

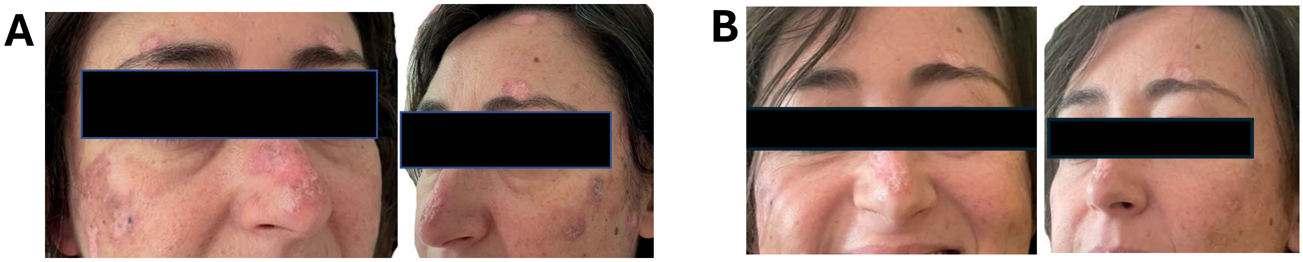

Supplementary Figure S1. Skin manifestations on the face of case 1 (A) before and (B) 4 weeks after the start of ECP therapy.

Table S1. Lipid levels and biochemical values at different time points of ECP treatment.

Laboratory parameters	Time points		
	Baseline	Week 3	Week 4
**Total Cholesterol mg/dL**	225,0	233,0	202,0 (-10.2%)
**LDL-Cholesterol mg/dL**	177,0	180,0	154,0 (-13%)
**Apolipoprotein B mg/dL**	143,2	150,3	122,1 (-14.7%)
**Lipoprotein(a) mg/dL**	15,3	13,7	12,6 (-17.6%)
**Triglycerides mg/dL**	80,0	111,0	74,0 (-7.5%)
**Apolipoprotein A1 mg/dL**	164,0	183,4	143,5 (-12.5%)
**HDL-Cholesterol mg/dL**	32,0	31,0	34,0 (+6%)
**ALT mg/dL**	6,0	9,0	5,0
**AST mg/dL**	9,0	11,0	7,0
**GGT U/L**	16,0	13,0	19,0
**Creatinine mg/dL**	0,6	0,5	0,5
**Glucose mg/dL**	83,0	73,0	149,0
